# 1903. Factors associated with response to a phone-administered alcohol and substance use survey during the COVID-19 pandemic among women in the MACS/WIHS Combined Cohort Study: Who are we missing?

**DOI:** 10.1093/ofid/ofac492.1530

**Published:** 2022-12-15

**Authors:** Hannah R Tierney, Yifei Ma, Peter Bacchetti, Ralph J DiClemente, Mirjam-Colette Kempf, Deborah L Jones, Lauren F Collins, Adaora A Adimora, Aruna Chandran, Audrey L French, Amanda B Spence, Jack DeHovitz, Anjali Sharma, Judith A Hahn, Jennifer C Price, Phyllis C Tien

**Affiliations:** University of California, San Francisco, San Francisco, California; University of California, San Francisco, San Francisco, California; University of California, San Francisco, San Francisco, California; NYU School of Global Public Health, New York, New York; University of Alabama at Birmingham, Birmingham, Alabama; University of Miami, Miami, Florida; Emory University, Atlanta, Georgia; University of North Carolina, Chapel Hill, North Carolina; Johns Hopkins University, Baltimore, Maryland; Stroger Hospital of Cook County, Chicago, Illinois; Georgetown University Medical Center, District of Columbia, District of Columbia; State University of New York-Downstate Medical Center, New York, New York; Albert Einstein College of Medicine, New York, New York; University of California, San Francisco, San Francisco, California; University of California, San Francisco, San Francisco, California; University of California, San Francisco, San Francisco, California

## Abstract

**Background:**

Early in the COVID-19 pandemic, many clinical studies pivoted to remote research visits, which have a higher non-response rate compared to in-person assessments. Survey non-response can bias estimates of alcohol and substance use prevalence. Our objective was to identify factors associated with responding to an alcohol and substance use phone survey administered during the COVID-19 pandemic to women enrolled in the MACS/WIHS Combined Cohort Study, a multicenter U.S. prospective cohort of adults living with and without HIV.

**Methods:**

We assessed associations of pre-pandemic (April-Sept. 2019) sociodemographic factors, HIV status, housing status, depressive symptoms, alcohol use, and substance use measures with response to a pandemic (Aug.-Sept. 2020) phone survey using multivariable logistic regression. Response probability weights generated from the regression model were applied to the sample and prevalence estimates of risky drinking (> 7 drinks/week or > 3 drinks/day) and substance use (opioids, stimulants, sedatives) in the COVID-19 pandemic were compared to the unweighted sample.

**Results:**

Of 1,834 women with pre-pandemic data, 62% were of Black race, 46% had an annual income < $12K, 71% were living with HIV and the mean age was 52.4 (SD 9.3) years. The phone survey response rate was 77.5%. In the adjusted model, the odds of responding were lower at research sites in the Western (aOR 0.35 95% CI 0.21-0.57) and Southern US (aOR 0.29 95% CI 0.19-0.44, ref=Midwest), in women of Hispanic ethnicity (aOR 0.47 95% CI 0.33-0.66, ref=Black race), and in those who reported substance use (aOR 0.62 95% CI 0.40-0.95). By contrast, the odds were higher for women of white race (aOR 1.63 95% CI 1.02-2.70) and those with stable housing (aOR 1.71 95% CI 1.22-2.39). Unweighted versus weighted prevalence estimates were 11.1% vs. 11.6% for risky drinking and 6.1% vs. 6.9% for substance use.

**Conclusion:**

Among a sample of socioeconomically disadvantaged women, women of Hispanic ethnicity, and those who were unstably housed and reported substance use at baseline had lower odds of responding to an alcohol and substance use phone survey conducted early in the COVID-19 pandemic. As remote survey methods become more common, investigators should ensure that data remain representative of the target population.

**Disclosures:**

**Ralph J. DiClemente, PhD**, Benten Technologies: Advisor/Consultant|Department of Defense: Advisor/Consultant|Morehouse School of Medicine: Advisor/Consultant|Northwell Health: Advisor/Consultant **Adaora A. Adimora, MD, MPH**, Gilead: Advisor/Consultant|Gilead: Grant/Research Support|Merck: Advisor/Consultant|Merck: Grant/Research Support **Anjali Sharma, MD, MS**, Gilead: Advisor/Consultant|Gilead: Grant/Research Support **Jennifer C. Price, MD, PhD**, Abbvie: Grant/Research Support|Gilead: Grant/Research Support|Merck: Grant/Research Support **Phyllis C. Tien, MD, MSc**, Gilead: Grant/Research Support|Merck: Grant/Research Support.

